# Towards reducing diagnostic delay in endometriosis in primary care: a qualitative study

**DOI:** 10.3399/BJGPO.2024.0019

**Published:** 2024-09-18

**Authors:** Laura de Kok, Henk Schers, Zoë Boersen, Didi Braat, Doreth Teunissen, Annemiek Nap

**Affiliations:** 1 Department of Obstetrics and Gynaecology, Radboud University Medical Center, Nijmegen, The Netherlands; 2 Department of Primary Care Medicine, Radboud University Medical Center, Nijmegen, The Netherlands; 3 Department of Obstetrics and Gynaecology, Rijnstate Hospital, Arnhem, The Netherlands

**Keywords:** Endometriosis, Primary care, Diagnostic delay

## Abstract

**Background:**

Due to a heterogeneity of symptoms, a lack of an adequate diagnostic test, and a lack of awareness, diagnostic delay in endometriosis in primary care on average amounts to 35 months.

**Aim:**

To determine which interventions are most feasible to reduce time to diagnosis in primary care, focusing on GPs’ preferences, the intervention’s content, design, and implementation.

**Design & setting:**

We conducted a qualitative study by performing focus groups with GPs and GP trainees between July and October 2021.

**Method:**

Data collection was continued until saturation was obtained. Focus groups were transcribed and openly encoded. Themes were formulated by three independent researchers.

**Results:**

Divided over five focus groups 22 GPs and 13 GP trainees participated. Three themes were formulated: increasing awareness, combined intervention, and reaching unaware GPs. Suggestions for a combined intervention strategy were adaptation of guidelines, a diagnostic support tool, and compulsory education. To reach unaware GPs, participants felt that education should be offered in regional networks and education for GP trainees should be mandatory. A guideline on menstrual symptoms should be considered and the term endometriosis should be added to the differential diagnosis paragraphs of existing guidelines. A diagnostic support tool should be linked to a guideline and consist of a flowchart with steps starting with the first presentation of symptoms leading to the diagnosis of endometriosis.

**Conclusion:**

According to GPs, a combined intervention strategy consisting of an adapted guideline, a diagnostic support tool, and education might be successful interventions in reducing diagnostic delay in endometriosis.

## How this fits in

A considerable share of diagnostic delay in endometriosis in the Netherlands occurs in primary care. In previous studies, barriers in the diagnostic process appeared to be a limitation in knowledge and awareness, the lack of appropriate guidelines, and insufficient collaboration between GPs and gynaecologists. Based on the findings of this qualitative study, we concluded that according to GPs, the delay in diagnosing endometriosis in primary care can be reduced by increasing awareness through a combined intervention strategy consisting of education, guideline adaptation, and a diagnostic tool with special attention to reach unaware GPs.

## Introduction

Endometriosis is a common condition that affects approximately 2–10% of fertile-aged women. It is defined as the presence of endometrium-like tissue outside the uterus, which induces a chronic, inflammatory reaction, causing a wide variety of clinical symptoms.^1^ Dysmenorrhoea, dyspareunia, and chronic pelvic pain are frequently mentioned complaints that have a negative effect on quality of life and social wellbeing, comparable with the effects of Crohn’s disease.^2,3^ Endometriosis not only impairs quality of life, but also affects the ability to work, causing a serious economic burden.^4^

Diagnosing endometriosis can be difficult. Despite the fact that some endometriosis related symptoms are very specific, there is often a wide heterogeneity of symptoms. Awareness among patients, GPs, and medical specialists, as well as sociocultural factors about treating pain are important factors in diagnosing endometriosis. In multiple studies, a long period of time, ranging from 6.7 to 11.7 years, is described between the onset of symptoms and diagnosis.^4–8^ This diagnostic delay can lead to suboptimal care that could then result in worse quality of life for women ultimately diagnosed with the disease, as well as higher healthcare utilisation and costs.^1,9^ It is therefore of the utmost importance to reduce the time to diagnosis.

GPs play a pivotal role in healthcare systems in different countries, including the Netherlands, as gatekeepers to secondary care, which means that a visit to a medical specialist in a hospital is only covered by insurance with a referral from the GP. As a consequence, women with symptoms will first present to the GP. It is therefore crucial that, if pragmatic treatment of symptoms is not successful, GPs recognise possible endometriosis, so they can aim at confirming the diagnosis or refer in a timely manner when necessary. The median diagnostic delay was estimated at 35 months in 2014.^8^ According to patients, GPs, and gynaecologists, barriers in this diagnostic process appeared to be a limitation in knowledge and awareness, the lack of appropriate guidelines, and insufficient collaboration between GPs and gynaecologists.^10–12^

A Delphi study, involving an expert team consisting of both different professionals in the field of endometriosis and GPs, provided ideas on interventions to reduce diagnostic delay in primary care. The proposed interventions included the development of more education, both in the curriculum for GP trainees and postacademic education for GPs, the development of a guideline and the development of decision support tools for use during consultations (Van der Zanden *et al*, personal communication, 2018).

Based on the finding that diagnostic delay in primary care is unacceptably long,^8^ we aimed to identify feasible interventions that target GPs and their role in the diagnostic process. We therefore conducted a qualitative study in collaboration with GPs to determine which interventions are, according to them, most feasible to reduce diagnostic delay in primary care, thereby focusing on GPs’ preferences and the intervention’s content, design, and implementation options.

### Methodology

We conducted a qualitative study because this provided us the opportunity to get in-depth data. We expected the interaction and discussion between participants during focus group interviews would lead to new insights and topics for further discussion. Focus group interviews with GPs and GP trainees were conducted between July and October 2021, until data saturation was reached, defined as no new information presented by participants in subsequent sessions, followed by one more session for confirmation. Because of the COVID-19 pandemic, only one session was held physically, while the remaining sessions were organised in the form of online video conferences.

We included a diverse group of participants, defined as GPs, GP trainees, academic and non-academic GPs, female and male GPs, GPs from geographically different parts of the country, and GPs with a different number of years of experience. By aiming at including a diverse group of participants, we were confident that we have included a reliable representation of the Dutch GPs. In the Netherlands, GPs can choose to undertake an additional training in an area of their special interest. One of those areas is the urogynaecology, which includes women’s health. We therefore aimed to include both GPs with and without special interest in urogynaecology. All GPs affiliated with the Radboudumc Academic Network received an email with an invitation to join a focus group on one of the proposed dates. Furthermore, we used social media to reach non-academic GPs and GP trainees from different regions in the Netherlands. Finally, we asked one group of GPs and one group of GP trainees to perform a focus group interview during one of their compulsory education sessions, to make sure that not only GPs with interest in endometriosis would participate. Several weeks prior to the meeting we informed them about this plan and asked them permission. These two groups consisted of a fixed composition. The other groups were composed depending on which dates the GPs and GP trainees were able to participate.

The focus group interviews were semi-structured, so participants could brainstorm freely with guidance from the moderator. The research team consisted of two gynaecologists (AN, DB), both professors in reproductive medicine and one of them specialised in endometriosis care, two GP researchers (HS, TT), of which one is professor in primary care and one specialised in urogynaecology, and two medical researchers in gynaecology (LK, ZB). During the focus group discussion a topic guide was used (Supplementary Appendix 1), compiled by three authors (LK, AN, HS) on the basis of literature and clinical experience. The topic guide was updated after each session. Specific topics were added in response to the first focus group sessions in order to discuss these options with the following groups. During the discussion in the first focus group, we experienced that participants had many substantive medical questions about endometriosis. This hampered the discussion about improving diagnostic delay. Because of this, we provided more clinical background information prior to the next focus groups before starting the discussion, to make sure that optimal use could be made of the time available. All focus group interviews were moderated by one researcher (LK) with assistance of one of two other researchers (AN or HS) to take notes and monitor the process. In the larger groups, both the moderator and the assistant moderator were attentive to ensure that all participants were heard equally. The focus group interviews were audio recorded and the online focus group interviews were also video recorded. Next, the recorded interviews were fully transcribed by one of the researchers (LK). In addition to the data from the focus group interviews, all participants completed a short questionnaire reflecting on their personal and professional situation. All participants signed an informed consent form.

**Figure 1.**
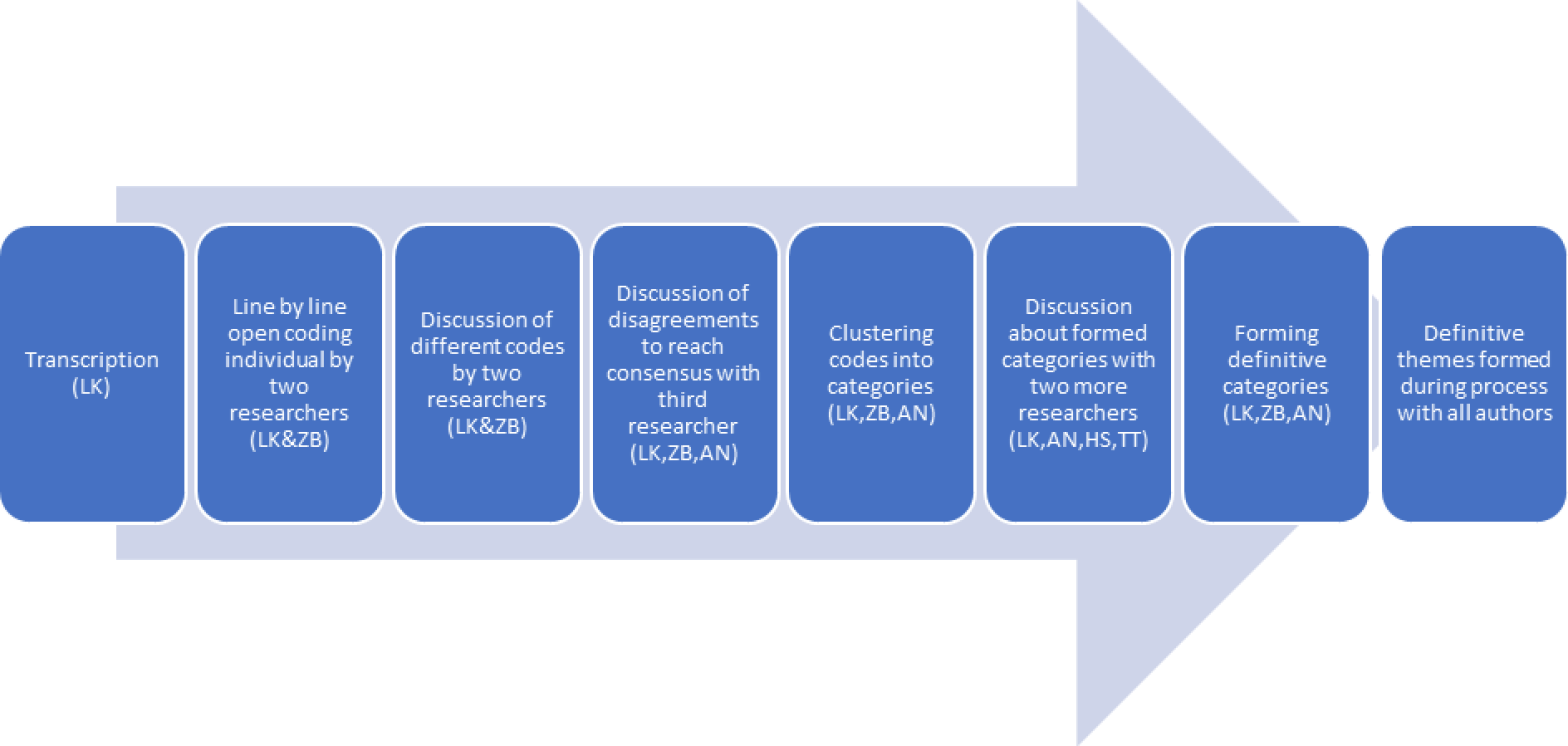
process of data analysis.

ATLAS-ti (version 9) was used, with thematic analysis, grounded theory methodology, applied as method for data analysis (Figure 1).^13,14^ The consolidated criteria for reporting qualitative research (COREQ-criteria) were applied to this manuscript. We used a quantification to indicate whether few (1–3), some (4–8), many (9–17), or most (18 or more) participants made a certain statement.^10,12,15^

## Results

Saturation was reached after five focus group interviews, which included a total of 35 participants (Table 1). The participants worked in practices divided over five provinces in the Netherlands. The number of years of experience of the GPs varied between two and 30 years. The duration of the focus group interviews varied between 49 and 88 minutes.

**Table 1.** Characteristics of participants per focus group interview.

	Total	Focus group 1	Focus group 2	Focus group 3	Focus group 4	Focus group 5
Number of participants	35	5	11	10	6	3
**Gender**						
Male Female	9 26	1 4	3 8	2 8	2 4	1 2
**Mean age, years**						
GP GP trainee	47.6 29.9	49.4	32.9	46.7	37.5	41.7
**Profession**						
GP GP trainee	22 13	5 0	1 10	10 0	3 3	3 0
**Location**						
Urban Rural Urbanised	12 8 15	1 1 3	7 1 3	0 4 6	3 2 1	1 0 2
**Type of practice**						
Solo Duo Group Health centre	2 10 15 8	0 1 3 1	1 2 5 3	0 4 6 0	1 1 1 3	0 2 0 1
Specialty training urogynaecology	3	1	0	1	0	1

Few participants stated that there was no need for an intervention, because they believed that early detection of endometriosis was not necessary. However, most participants had the opinion that initiating one or more interventions could improve time to diagnosis. During the process of analysis, we divided the data into four formulated categories, based on grouping of the different codes: preferences, design, content, and implementation (Table 2). After another discussion with the entire research team, three overarching themes emerged from these categories: increasing awareness, combined intervention, and reaching unaware GPs.

**Table 2.** Overview of categories and corresponding codes with number of quotations.

**Preferences**	(Adaptation of existing) guideline	45
	Education for GPs and/or GP trainees	16
	Combination of guideline with education and/or diagnostic decision tool	12
	Diagnostic decision support tool	8
	No need for any intervention	2
**Design**	**General**	
	Easily accessible	14
	Ready-made package	13
	Not too extensive	4
	**Education**	
	Provided by gynaecologist or GP with special interest in urogynaecology	40
	In regional networks of GPs with obligatory educational meetings (initiated by GPs)	17
	Clinical examples of individual cases	17
	E-learning together with guideline	12
	Regularly repeating pattern of education options	5
	**Guideline**	
	Include endometriosis in differential diagnostic chapters of existing guidelines	20
	**Diagnostic decision support tool**	
	Should be added to guideline	11
	Should not be an app	6
	Has to be evidence based	2
**Content**	**Subject of intervention**	
	Gynaecological causes of (chronic) pelvic pain	25
	Menstrual complaints	20
	Intervention specific on endometriosis	1
	**Topics to address**	
	Treatment in primary care and moment of referral	26
	Physical and/or gynaecological examination	16
	Clinical background information such as risk factors, age, normal menstruation, amount of blood loss and pain	12
	Anamnestic questions	10
	Diagnostic procedure	9
**Implementation**	Investigation of effect by file research in primary care	4
	Investigation of effect by research number of referrals	2
	Investigation effect of education by exams	1
	Implementation by publishing guideline on specific website	3
	Implementation of guideline by publishing in Dutch journal	2
	Implementation of guideline by standardised process	1

### Increasing awareness

Most participants reported that increasing awareness is an important step in reducing diagnostic delay and that reaching those GPs who are not aware of the problem during consultation constitutes a challenge for the development of an intervention:

'*If you’re unconsciously incompetent in this area, then you just don’t know there is a gap, so how are you going to find out where the unconsiously incompetent people are and how do you make them more consciously incompetent, to eventually make them more aware and consiously competent*,' (GP, female, 31 years)

Many participants expressed the concern that education would only reach a small group of GPs who are already interested in the topic. Moreover, some GPs had the opinion that a diagnostic aid about endometriosis as well as a guideline on endometriosis would only be useful for those GPs who are already aware of the possibility of the diagnosis.

In the context of increasing awareness, participants also discussed the content of an intervention. They discussed whether it should be specifically diagnosis-based containing information on endometriosis or problem-based with information on pelvic pain or menstrual complaints. The perceived disadvantage of an intervention that was substantively focused on endometriosis was that awareness during consultations would probably not be increased. However, according to the participants, an intervention based on a complaint could become too extensive:

*'It also seems difficult to me to get a complete overview of all pelvic pain complaints in one diagnostic schedule, which also really works well in* [general] *practice, because there is so much more than just gynaecology*,' (GP trainee, female, 30 years)

### Combined intervention strategy

**Figure 2.**
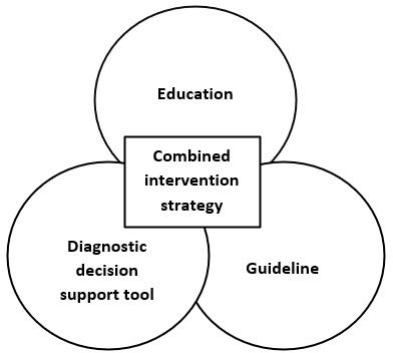
Combined intervention strategy.

Many participants stated that a combination of interventions would be the best option to reduce diagnostic delay (Figure 2). According to them, this combined strategy should involve education, a decision support tool, and the development of a guideline.

'*I think you achieve that by paying attention to it in training, so people start thinking "*oh yeah*", and then next time they might even check out a guideline*,' (GP, female, 33 years)

Many GPs indicated that education for GP trainees is an important intervention and some GPs preferred postgraduate education involving clinical examples and gynaecological causes of pelvic pain.

Some GPs preferred a diagnostic decision support tool that can help find a diagnosis for women with pelvic pain. They explained that this tool should be a flowchart containing endometriosis-associated symptoms, questions that can be asked about these symptoms and physical examination, resulting in a recommendation about endometriosis being less or more likely, pragmatical treatment, and whether or not to refer the patient. Many GPs stated that a diagnostic decision support tool should be easily accessible and that this could be achieved by including the tool in a guideline. According to few participants, a tool should not be too extensive in order to retain its usability. Few GPs pointed out that it is important that this tool is evidence-based. After having discussed the possibility of designing a diagnostic decision support app, some participants reported that they would not opt for an app because there are already a lot of other apps available, but they never use them.

*'I think that it* [diagnostic aid] *shouldn’t be too long or too hard, and indeed be implemented in a guideline*,' (GP, female, 41 years)

Most GPs preferred more attention for endometriosis in the guidelines of the Dutch College of GPs (NHG). They indicated that adapting an existing guideline, for example the guideline on vaginal bleeding, would be preferred above developing a new one, because there are already many different guidelines. Most participants preferred a guideline on menstrual complaints, whereas some preferred a guideline on gynaecologic causes of pelvic pain.

Three substantive topics emerged in all focus groups that, according to the participants, would have to be addressed in the above-mentioned combined intervention strategy. These topics are adequate anamnestic questions, diagnostic tests, and therapeutic options. Participants indicated that, with regard to anamnestic questions, pain and amount of blood loss during menstruation should be discussed. According to the participants, diagnostic testing should include a physical gynaecological examination. Some mentioned that extra attention should be paid to conceivable practical barriers in general practice, such as perceived lack of time and the lack of a suitable chair for gynaecological examination. Moreover, according to participants, information should be provided on the limited value of a negative ultrasound. Therapeutic options should include pragmatic treatment with analgesics or oral contraceptives and indications for referral to a gynaecologist.

*'I think it would be really good to know for us GPs, what to treat yourself, what complaints should be treated by ourselves, and when do you reach the point for additional diagnostics, are you going to request an ultrasound easily for example, to get some certainty for the patient on the diagnosis, or just treat blindly, I think those are all questions which could be overcome in a menstrual complaints guideline, but also in an pelvic pain guideline, to create more awareness*,' (GP trainee, female, 29 years)

Regarding the effect of the combined intervention strategy, few participants stated that the success of its implementation could be tested by measuring the total number of referrals to a gynaecologist with the specific suspicion of endometriosis. Moreover, this could be measured by qualitative analysis of patient records in general practices.

*'That might be quite nice to do, a baseline measurement, so that you do look indeed how long it takes at this moment before people are being diagnosed with endometriosis and that you also, maybe the baseline measurement is quite disappointing, that you indeed see that it is such a long delay and that you then look after a few years or a certain time later*,' (GP, male, 59 years)

### Reaching unaware GPs

To reach GPs who are not interested in endometriosis beforehand, most participants suggested that education should be offered in regional networks of GPs for whom educational meetings are obligatory:

*'Yes, I share the comment that people who are interested will do a training in such an area, but you will miss the rest. I was thinking about that for a while. I think that if you can fit a therapeutical part about hormonal treatment in an FTO* [a mandatory meeting for regional GP groups], *you will reach a whole new group of colleagues*,' (GP, male, 43 years)

Many participants reported that these meetings should be organised by GPs, and that they would benefit from a ready-made training package designed by gynaecologists or GPs with special interest in urogynaecology. Another way to reach more GPs, mentioned by some participants, was a multi-day training with various topics, one of which being endometriosis. Moreover, according to some GPs, education should be interactive and repeated once every 5 years. Many participants would prefer a module in a national e-learning package developed by the NHG, accompanying a guideline to reach a large group of GPs.

Many participants indicated that education for GP trainees should be provided by gynaecologists or by GPs with special interest in urogynaecology. According to some this should be integrated as a mandatory part in the curriculum to reach all of them.

Most of the participants would like to see the term endometriosis in the differential diagnosis chapter of other guidelines, such as the guideline on irritable bowel syndrome (IBS), so they would be aware earlier in case they suspect patients with pelvic pain of other diagnoses.

'*Yes, that IBS guideline that you refer to, in my opinion it would be suited for that, that already does exist. Because I think, for me, I don’t need that much in a guideline, except to be reminded of endometriosis, because almost every one of us already knows what you are telling, or we actually do know all of it, but I hardly ever think of it, while if it’s mentioned somewhere in a line and I will see that, following the guideline, I will start thinking of endometriosis and that would do it for me*,' (GP trainee, male, 30 years)

## Discussion

### Summary

In this study, we investigated GPs opinions about interventions that may lead to a reduction of the diagnostic delay in patients with endometriosis in primary care. In order to find acceptable interventions, we created a multidisciplinary research group by which a qualitative study was designed. The main outcome was that GPs stated that it is of great importance to develop, validate, and implement a useful and effective intervention to reduce diagnostic delay in primary care and that awareness should be increased by a combined intervention strategy. This strategy should include offering education in an obligatory manner, the development of a diagnostic decision support tool, and adaptation of an existing GP guideline by adding information on endometriosis. Moreover, to reach unaware GPs, education should be offered in regional networks and/or in multi-day training in which different topics are addressed. For GP trainees, education should be included in the mandatory part of the curriculum. An education package for GP trainees and postacademic training for GPs could be helpful. Furthermore, a diagnostic decision support tool should be developed. This tool should consist of a flowchart, starting with the presentation of symptoms leading to the diagnosis of endometriosis and treatment options. Finally, in an already existing GP guideline, information should be added about menstrual complaints and endometriosis-associated symptoms, and the term endometriosis should be added to the differential diagnosis chapters of other guidelines. The diagnostic decision support tool should be linked to the adapted guideline on menstrual complaints.

### Strengths and limitations

The most important strength of this study is its qualitative character. Because of this, new ideas and insights evolved during the focus group sessions with GPs. Awareness during everyday consultations emerged as an important theme and several suggestions were made on how to increase this. Furthermore, the idea of combining interventions, instead of choosing one, arose during the focus group interviews. Finally, the importance of unaware GPs arose during the focus group sessions and specific ideas about how to reach them emerged from the discussion. These outcomes would not have appeared in a questionnaire study.

Another strength of our study is that we used a rigorous, reproducible, and transparent design. Data were collected in a reproducible manner and processed transparently by two independent researchers, and all steps of data analysis were discussed within the complete research team.

Finally, a strength of the study is that we created sample diversity, resulting in a high variety of opinions. A substantial part of the participants were GP trainees, the GPs were geographically spread over different provinces in the Netherlands, GPs from different types of practices participated, and both men and women were included, with a majority of women, which is consistent with the composition of GPs in daily practice in the Netherlands.

A possible limitation of this study is that GPs and GP trainees with a special interest in gynaecology might be more willing to participate in our study. Interestingly, one of the outcomes of our study was that it may be a challenge to reach unaware GPs. We minimised this risk by asking one fixed GP peer group and one fixed group of GP trainees to join this study.

Another limitation lies in the fact that most focus group interviews had to be conducted online because of the COVID-19 pandemic and only one meeting was held physically, which caused a heterogeneity in data collection that we had not foreseen. However, in the literature no disadvantages of online focus group interviews compared to conventional meetings have been described and the amount of data to collect from the focus group interviews is equal.^16,17^ We are therefore confident that the outcomes of this study have not been influenced by this heterogeneity in data collection.

### Comparison with existing literature

Barriers in the diagnostic process in patients with endometriosis have been studied before. However, to our knowledge a study, designed by GPs and gynaecologists, including GPs to identify specific intervention strategies that could be used by GPs has not been conducted before.

A recent qualitative study conducted on GPs in the UK explored what GPs identified as important considerations when caring for women with symptoms suspect for endometriosis.^18^ This important study revealed the complexity of the diagnostic process and described many valuable issues raised by GPs for the delay in diagnosis, such as the heterogeneity of symptoms, the need to follow a clinical hierarchy of diagnoses, concerns about laparoscopy, the lack of awareness, and lack of education. Another important issue raised by GPs is the question whether diagnosis is needed. If adequate symptom control is achieved by pragmatic treatment using analgesics or hormonal therapy, a further diagnostic process may not be indicated. However, if pragmatic treatment is not effective, a diagnosis should be the aim. A careful consideration of the balance between risks and benefits of a diagnostic process is necessary. In this process, it is of utmost importance to discuss with patients what their preferences are using shared decision-making. In the study, potential explanations for the delays in diagnosis were identified, but no targeted interventions were proposed. The importance of awareness is evident, both in this study and our study. A preliminary clinical diagnosis could suffice, but if pragmatic treatment of symptoms fails, GPs should consider endometriosis as a diagnosis. In order to consider this diagnosis, GPs have to be aware of it. Therefore, adequate interventions that are useful to healthcare professionals in primary care are needed. The interventions proposed by GPs in our study are in accordance with the described explanations for diagnostic delay by GPs in the UK.

A French questionnaire study showed that both levels of knowledge and awareness are low among GPs, however the are higher in GPs with a university diploma in gynaecology or participating in continuing medical education in gynaecology. This study therefore concludes that the diagnostic delay should be reduced by better medical training for GPs.^19^ In our study, education is also seen by GPs as an important component of the combined intervention strategy. In addition, GPs offered specific proposals about the design and the content of education, as well as implementation options. Implementing guidelines and innovations in general practice have been subject to research before. A review showed that multifaceted interventions are more effective in inducing change in general practice, which corresponds to our results of a proposed combined intervention strategy.^20^

### Implications for research and practice

In this qualitative study, we aimed to determine GPs opinions about a feasible intervention strategy to reduce the time to diagnosis of endometriosis in primary care. In our opinion, the combined intervention strategy that was proposed by GPs should be developed and implemented in the Netherlands. The research team believes that the specific ideas of an education package for postacademic education in regional networks, education for GP trainees in the mandatory part of the curriculum, a decision support tool linked to a guideline, and adding the term endometriosis to differential diagnosis chapters of existing guidelines are feasible and useful interventions. However, most GPs stated that an existing guideline, for example the guideline about vaginal bleeding, should be adapted by adding information about endometriosis. In contrast, we would recommend to develop a specified guideline about menstrual symptoms, and not just addressing endometriosis, because symptoms can be treated in primary care. If pragmatic treatment is not successful, GPs can aim at confirming the diagnosis or refer in a timely manner when necessary. Another point of critical reflection is that GPs mentioned that the success of implementation could be tested by measuring the total number of referrals of patients to the hospital. In our opinion, number of referrals is no reliable instrument to measure the success of implementation. Instead, asking the patient about diagnostic delay may be a better way to investigate implementation. Moreover, the current study was developed to design one specific intervention to implement. We agree with the GPs that a combined intervention strategy may be better to reduce diagnostic delay, however in practice this can be more complex and expensive to implement. In a future study, in collaboration with GPs, patients, and gynaecologists, we will explore the possibility of developing and implementing the proposed combined intervention strategy, consisting of a guideline about menstrual symptoms and a decision support tool and education for GP and GP trainees.
